# Neuron-Specific Regulation of Associative Learning and Memory by MAGI-1 in *C. elegans*

**DOI:** 10.1371/journal.pone.0006019

**Published:** 2009-06-24

**Authors:** Attila Stetak, Frederic Hörndli, Andres V. Maricq, Sander van den Heuvel, Alex Hajnal

**Affiliations:** 1 Institute of Zoology, University of Zürich, Zürich, Switzerland; 2 Department of Developmental Biology, Utrecht University, Utrecht, The Netherlands; 3 Department of Biology, University of Utah, Salt Lake City, Utah, United States of America; L'université Pierre et Marie Curie, France

## Abstract

**Background:**

Identifying the molecular mechanisms and neural circuits that control learning and memory are major challenges in neuroscience. Mammalian MAGI/S-SCAM is a multi-PDZ domain synaptic scaffolding protein that interacts with a number of postsynaptic signaling proteins and is thereby thought to regulate synaptic plasticity [1], [2], [3].

**Principal Findings:**

While investigating the behavioral defects of *C. elegans* nematodes carrying a mutation in the single MAGI ortholog *magi-1*, we have identified specific neurons that require MAGI-1 function for different aspects of associative learning and memory. Various sensory stimuli and a food deprivation signal are associated in RIA interneurons during learning, while additional expression of MAGI-1 in glutamatergic AVA, AVD and possibly AVE interneurons is required for efficient memory consolidation, i.e. the ability to retain the conditioned changes in behavior over time. During associative learning, MAGI-1 in RIA neurons controls in a cell non-autonomous fashion the dynamic remodeling of AVA, AVD and AVE synapses containing the ionotropic glutamate receptor (iGluR) GLR-1 [4]. During memory consolidation, however, MAGI-1 controls GLR-1 clustering in AVA and AVD interneurons cell-autonomously and depends on the ability to interact with the β-catenin HMP-2.

**Significance:**

Together, these results indicate that different aspects of associative learning and memory in *C. elegans* are likely carried out by distinct subsets of interneurons. The synaptic scaffolding protein MAGI-1 plays a critical role in these processes in part by regulating the clustering of iGluRs at synapses.

## Introduction

Dynamic changes involving the formation of new synapses, morphological changes of dendrites, increased numbers of dendritic spines and the redistribution of synaptic proteins create the remarkable plasticity of the nervous system [5], [6]. Activation of neurotransmitter receptors on the postsynaptic side of the synapse also triggers biochemical pathways that lead to changes in neuronal function. NMDA and AMPA subtypes of ionotropic glutamate-receptors (iGluR) play crucial role in vertebrate synaptic plasticity, and are linked by membrane-associated guanylate kinases (MAGUKs) into large signaling complexes. However, the *in vivo* role of MAGUKs in LTP formation and learning is largely unknown. Mammalian MAGI/S-SCAM is a postsynaptic multi-PDZ domain-containing MAGUK with a unique inverted domain organization [2]. MAGI was reported to interact with a multitude of neuronal proteins including NMDA receptors, transmembrane AMPA receptor regulating proteins (TARPs), a number of other neuronal proteins, and β-catenin [1], [7], [8], [9]. Therefore MAGI proteins are thought to play an important role in synaptic plasticity and memory formation.

The relatively simple nervous system of *C. elegans* composed of 302 neurons allows non-associative (adaptation and habituation) as well as associative learning between a variety of volatile or soluble chemoattractants, or cultivation temperature, and food. In order to investigate associative learning in *C. elegans*, several learning assays have been established, for example, salt chemotaxis learning [10], olfactory associative learning [11], [12], and temperature learning [13], [14]. Previous studies have also shown that regulators of learning and memory are conserved between mammals and *C. elegans* [11], [12], [15], [16]. AMPA type of ionotropic glutamate receptors not only play a critical role in vertebrate synaptic plasticity, but glutamate neurotransmission has also been shown to be involved in habituation of the tap withdrawal response as well as in olfactory associative learning in *C. elegans* [12], [16]. Therefore, the analysis of genes found in *C. elegans* can provide important insights into the mechanisms of learning and memory in vertebrates including humans.

Vertebrate genomes encode three MAGI isoforms, while a single MAGI-1 ortholog exists in *C. elegans*. Therefore, *C. elegans* is an ideal model to study the function of the MAGI-1 protein in associative learning and neural plasticity. In this study, we generated a deletion mutant of *magi-1* and investigated the learning and memory defect in the mutant worms. We found that loss of *magi-1* function, regardless of the sensory input, impairs associative learning in *C. elegans*. Expression of the wild-type MAGI-1 protein in a single pair of interneurons can rescue the learning defects. In addition, *magi-1* mutant worms show defects in memory consolidation, which requires the wild-type MAGI-1 protein in a distinct set of interneurons. We also found that MAGI -1 controls the distribution of ionotropic glutamate receptor, GLR-1 during learning and memory consolidation. We propose a molecular mechanism for MAGI-1 during neural plasticity, which modulatory function may be conserved in mammals.

## Results

### Loss of MAGI-1 function impairs associative learning independent of sensory input

In order to study MAGI function in a simple animal model, we generated a knockout allele (*zh66*) of the single *C. elegans* MAGI ortholog *magi-1* (Fig. S1b). The *magi-1* locus encodes two mRNAs that are transcribed from two alternative promoters (Fig. S1a, b). The *magi-1(zh66)* deletion allele removes most of the region common to both isoforms. Total protein extracts from *zh66* animals lacked both isoforms as determined by Western blot analysis (Fig. S1c). Thus, the *zh66* deletion likely represents a *magi-1* loss-of-function *(lf)* allele. *magi-1(lf)* mutants appear healthy, fertile and display no obvious morphological or locomotory defects (data not shown).

**Figure 1:**
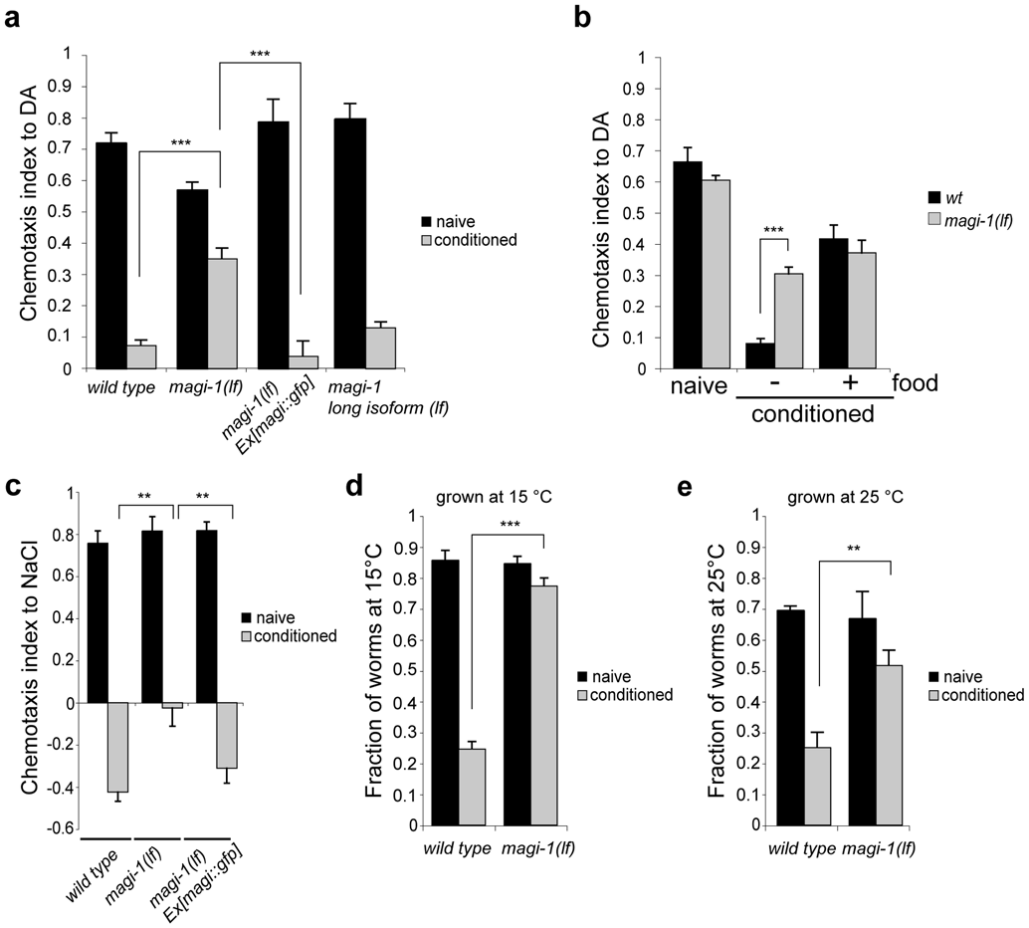
MAGI-1 is essential for associative learning. a, Context-dependent starvation conditioning of wild-type or mutant worms was assayed towards 0.1% DA volatile chemo-attractant without (naïve) or with (conditioned) pre-incubation with DA. Chemotaxis index was calculated as CI = (worms at DA - worms at solvent)/total number of worms. b, Chemotaxis towards DA was tested in the presence or absence of food. c, Associative learning of *magi-1(lf)* and wild-type animals by NaCl starvation conditioning. d and e, *magi-1(lf)* animals were tested in a thermotaxis association paradigm by starvation conditioning the animals at 15°C or 25°C. All experiments were done in triplicate and repeated at least three times. Error bars indicate average±S.E.M. Significance between datasets was tested with two-tailed student's t-test (** p<0.01, *** p<0.001).

To investigate a potential role of MAGI-1 in *C. elegans* olfactory associative learning, we first tested the chemotaxis of *magi-1(lf)* animals towards different compounds. The chemotaxis of *magi-1(lf)* mutants to six different volatile attractants, one soluble attractant, and a repellent was comparable to the response of the wild-type N2 strain (Fig. S2a). Furthermore, both wild-type and *magi-1(lf)* mutants responded similarly to food starvation, indicating that *magi-1(lf)* mutants have no obvious sensory or motor defects (Fig. S2b). To test the role of MAGI-1 in associative learning, we used an established context-dependent starvation conditioning protocol [14], [17]. In the olfactory learning assay, unconditioned wild-type and *magi-1(lf)* animals both exhibited strong chemotaxis towards diacetyl (DA) (Fig. 1a). However, after a one-hour starvation period in the presence of DA, wild-type animals displayed a strongly reduced attraction to DA, whereas starvation-conditioned *magi-1(lf)* mutants exhibited only a partial reduction in chemotaxis towards DA (Fig. 1a). We obtained similar results when using isoamyl alcohol as the attractant (data not shown). The *magi-1(tm446)* deletion that removes only the long isoform, on the other hand had no significant effect on olfactory learning (Fig. 1a). This result suggests that the two MAGI-1 isoforms act in a redundant manner during associative learning. To exclude the possibility that the conditioning defect in *magi-1(lf)* mutants was due to changes in adaptation rather than association, we conditioned *magi-1(lf)* mutants and wild type controls with DA in the presence of abundant food (Fig. 1b). In this experimental context, *magi-1(lf)* mutants were not defective in DA adaptation when compared to wild-type (Fig. 1b).

As a next step, we wondered if the observed associative learning defect was specific to volatile chemo-attractants or reflected a more general learning deficit. Indeed, *magi-1(lf)* mutants also showed strong defects in gustatory (salt chemotaxis) and thermotaxis conditioning assays. Starvation of wild-type animals in the presence of NaCl induced a strong avoidance of NaCl in subsequent chemotaxis assays (Fig. 1c). *magi-1(lf)* mutants, on the other hand, showed a greatly reduced avoidance of NaCl after starvation conditioning (Fig. 1c). In addition, *magi-1(lf)* animals failed to associate low (15°) or high (25°) temperatures with food starvation (Fig. 1d, e). We conclude that the loss of MAGI-1 function does not affect adaptation but impairs associative learning independently of the type of sensory input, as we found that MAGI-1 is required for the association of a food starvation signal with diverse environmental stimuli such as smell, taste or temperature.

### MAGI-1 acts in RIA interneurons during associative learning

**Figure 2:**
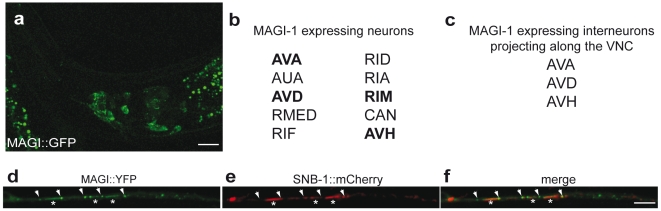
Expression pattern and sub-cellular localization of MAGI-1::GFP. a, expression of the rescuing *magi-1::gfp* construct in the head of an adult worm visualized by fluorescence confocal microscopy. b and c, list of the neurons expressing MAGI-1. MAGI-1 and GLR-1 double positive cells are indicated in bold. Neurons that project their processes along the ventral nerve chord are listed in c. d–f, co-localization of MAGI-1::YFP with the presynaptic marker, synaptobrevin (SNB-1) driven by endogenous promoters. Asterisks label the synaptic area, arrows point to the punctae flanking the synaptic cleft. Scale bars represent 10 µm on panel a, and 1 µm on panel f.

**Figure 3:**
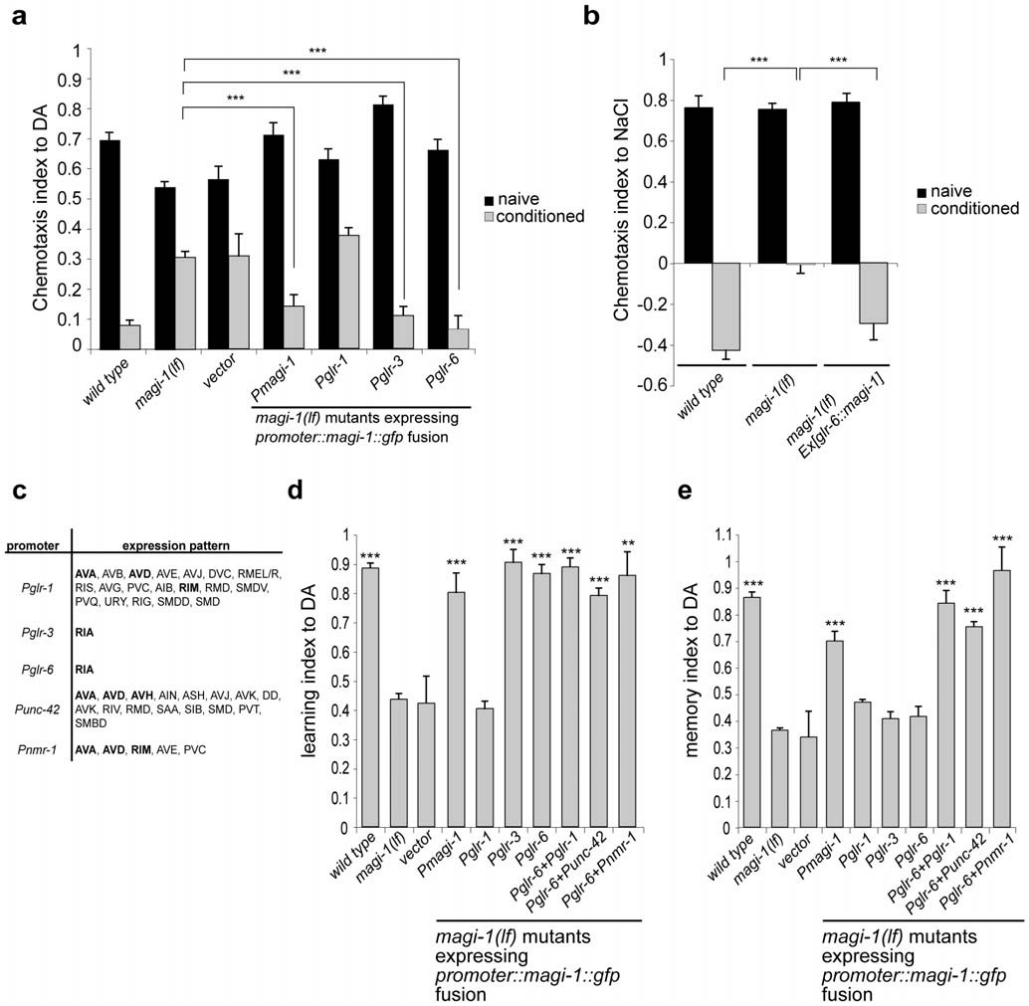
Associative learning requires MAGI-1 in RIA, while memory formation depends on MAGI-1 in AVA. Tissue-specific rescue of a, the olfactory associative learning defect and b, the gustatory (NaCl) learning defect of *magi-1(lf)* mutants. c, Expression pattern of the different neural promoters used in a, b, d and e. MAGI-1::GFP expressing neurons are highlighted in bold. d, *magi-1(lf)* mutant worms carrying empty vector, or the *magi-1::gfp* minigene under the control of different promoters were conditioned with DA and tested immediately. Learning index was calculated as LI = (CI_naïve worms_- CI_DA conditioned worms_)/CI_naïve worms_. e, Memory index of DA conditioned *magi-1(lf)* worms carrying empty vector or *magi-1::gfp* minigene under the control of different promoters following 30 minutes recovery in the absence of DA. Memory index was calculated as MI = (CI_naïve worms_- CI_DA conditioned and recovered worms_)/CI_naïve worms_. All experiments were done in triplicate and repeated at least in two independent experiments. Three independent transgenic lines were tested for each construct. Error bars indicate average±S.E.M. Datasets were compared to *magi-1(lf)* mutants using two-tailed student's t-test (** p<0.01, *** p<0.001).

**Figure 4:**
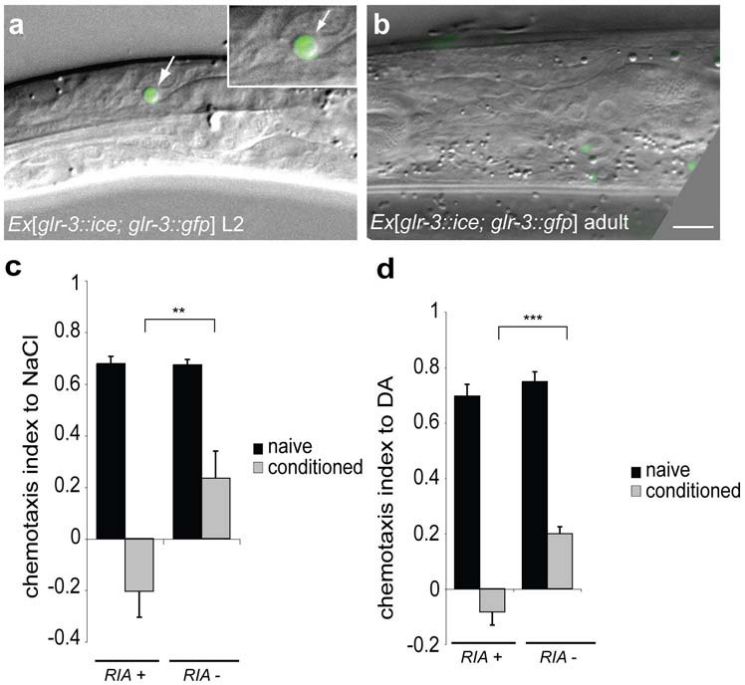
The RIA interneurons are required for associative learning. RIA was genetically ablated by expressing a *glr-3* promoter-driven activated ICE caspase. a, expression of the caspase in RIA (labeled with GFP) leads to apoptosis that is characterized by a refractile disc (see also inset) during larval development. b, 59.6% (n = 3200) of the adult worms lack RIA visualized by the missing *Pglr-3::gfp* reporter expression. Removal of the RIA neuron impairs learning both in c, salt and in d, olfactory associative learning paradigms. For quantitative analysis, three independent experiments were analyzed. Error bars indicate average±S.E.M. Significance between datasets as indicated was tested with two-tailed student's t-test (** p<0.01, *** p<0.001).

To study the MAGI-1 expression pattern, we generated a construct encoding the long isoform of *magi-1* fused to GFP (Fig. S1b). This *magi-1::gfp* minigene construct rescued the behavioral defects of *magi-1(lf)* mutants (Fig. 1a, c). MAGI-1::GFP was expressed in several interneurons, including AVA, AVD, AVE, RIM, and RIA (Fig. 2a, b). These neurons are all known to express ionotropic glutamate receptors (iGluRs) [4]. Therefore, we performed tissue-specific rescue experiments to determine the cellular focus of action. We tested empty pPD95.67 vector, or expressed the *magi-1::gfp* coding region under the control of a 2.7 kb fragment of the *glr-1* promoter, which is active in RIM, AVA, AVD and AVE, or the *glr-3* or *glr-6* promoters, which are exclusively active in RIA neurons (Fig. 3c). In the olfactory learning paradigm, *Pglr-3* or *Pglr-6*-driven *magi-1::gfp* rescued the learning defects of *magi-1(lf)* mutants, while no rescue was observed with *Pglr-1*-driven *magi-1::gfp* (Fig 3a,d). Expression of *magi-1* in RIA neurons also rescued the learning defect in the salt chemotaxis learning paradigm (Fig. 3b). Therefore, expression of *magi-1* in RIA was sufficient to restore associative learning to *magi-1(lf)* mutants in an immediate recall test. To further confirm that RIA plays a central role in associative learning, we ablated the RIA neuron using *glr-3* promoter-driven activated ICE caspase (Fig. 4a, b). In order to visualize the efficient ablation of RIA we co-expressed glr-3 driven GFP together with ICE caspase. Expression of the activated caspase led to apoptotic cell death of RIA neurons in early larvae in 59.6% of the transgenic animals (Fig. 4a, b). Transgenic worms where ICE activity was insufficient to kill RIA were used as internal controls. Genetic ablation of RIA neurons caused associative learning defects both in the volatile attractant and salt chemotaxis learning paradigms (Fig. 4c, d). Together with previous findings [15], these results confirm the central role of RIA neurons in a neural circuit mediating associative learning.

### Efficient memory consolidation requires MAGI-1 function in AVA and AVD interneurons

We next tested the memory consolidation, the capability of the animals to retain a conditioned behavior over time, by starvation conditioning the animals in the presence of DA and letting the animals recover for 30 minutes in the absence of DA before testing their chemotaxis [18]. In wild-type animals, the negative association of DA with starvation persisted during the 30-minute recovery period (Fig. 3e). Interestingly, although expression of *magi-1* in RIA neurons using the *glr-6* or *glr-3* promoters rescued conditioning in *magi-1* mutants (fig. 3d), it was not sufficient to restore memory consolidation over the 30 minutes recovery phase (Fig. 3e). Since MAGI-1 is not exclusively expressed in RIA, we speculated that memory consolidation might require MAGI-1 expression in additional neurons such as AVA, AVD, AVE, or RIM. We therefore co-expressed MAGI-1 under the control of different neuron-specific promoters (Fig. 3c) together with the RIA-specific *glr-6* promoter construct and tested the double transgenic animals for memory consolidation. Co-expression of *magi-1::gfp* under the *glr-6* promoter with either of the *glr-1*, *unc-42* or *nmr-1* promoters rescued the memory consolidation defects of *magi-1(lf)* mutants (Fig. 3d). Since no MAGI-1::GFP expression was detected in AVE and the *unc-42* promoter is not active in AVE and RIM, furthermore, AVE is mainly presynaptic in the ventral nerve chord, *magi-1* acts most likely predominantly in AVA and AVD during memory consolidation. However, our results cannot fully exclude that participation of additional neurons during memory consolidation are also required. AVA, AVD, and AVE interneurons express the GLR-1 glutamate receptor subunit [4], which has been shown to be essential for associative learning [16] as well as for the nose-touch response [19]. Similar to *glr-1(lf)* mutants, *magi-1(lf)* mutants showed a defect in their nose-touch response that could be rescued by expressing *magi-1::gfp* under the control of the *glr-1* promoter (Fig. S3), suggesting that MAGI-1 is required together with GLR-1 in AVA and AVD interneurons for a proper nose touch response. AVA, AVD and AVE neurons project their processes along the ventral nerve chord (VNC). GLR-1 localizes in AVA, AVD, AVE and other VNC synapses in punctae corresponding to receptor clusters in post-synaptic regions [20]. Furthermore, MAGI-1::YFP was localized in the VNC adjacent to the presynaptic marker synaptobrevin (SNB-1), most likely in the postsynaptic region as well as in distinct dots flanking the synaptic cleft (Fig. 2d–f). Thus, efficient memory consolidation requires MAGI-1 function in glutamatergic AVA and AVD and possibly in AVE interneurons.

### MAGI-1 regulates the remodeling of GLR-1 containing synapses during associative learning

**Figure 5:**
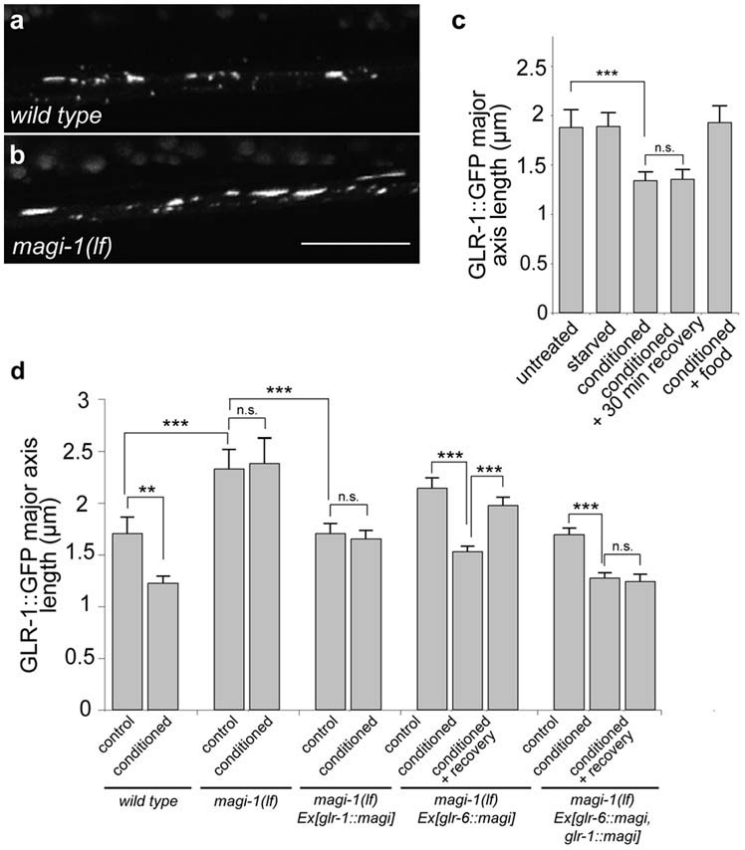
MAGI-1 regulates GLR-1 localization during associative learning and memory formation. Confocal images of the anterior half of the ventral nerve cord of a 4 days old adult a, Naïve wild-type and b, *magi-1(lf)* worms carrying the *glr-1::gfp* transgene c, Average size of GLR-1::GFP clusters in the posterior VNC upon starvation in the absence (starved, n = 174) or presence of 0.1% DA (conditioned, n = 181), conditioning followed by 30 minutes recovery in the absence of DA (conditioned+30 min recovery, n = 119), or conditioning in the presence of abundant food (conditioned+food, n = 84). d, Average size of GLR-1::GFP clusters in the posterior VNC upon starvation without (control) or with 0.1% DA (conditioned) or conditioning followed by 30 minutes recovery in the absence of DA (conditioned+recovery) in wild-type and *magi-1(lf)* animals carrying the indicated transgenes (wild type: control n = 80, conditioned n = 181; *magi-1(lf)*: control n = 86, conditioned n = 55; *magi-1(lf); Ex[glr-1::magi-1]*: control n = 182, conditioned n = 165; *magi-1(lf); Ex[glr-6::magi-1]*: control n = 202, conditioned n = 288, conditioned+recovery n = 186; *magi-1(lf); Ex[glr-1::magi-1, glr-6::magi-1]*: control n = 233, conditioned n = 271, conditioned+recovery n = 253). Three independent experiments were quantified, and three independent transgenic lines were tested for each construct. Error bars indicate average±S.E.M. Significance between datasets as indicated was tested with two-tailed student's t-test (n.s. p>0.05, ** p<0.01, *** p<0.001).

Since MAGI-1 function is required in AVA and AVD neurons for memory consolidation, we asked whether GLR-1 containing AVA and AVD synapses in the VNC posterior to the vulva are remodeled during associative learning using a functional *glr-1::gfp* reporter [20]. In wild-type animals, synaptic GLR-1::GFP cluster size in the posterior VNC remained unchanged after starvation or exposure to DA in the presence of abundant food alone (Fig. 5a, c) (naïve: 1.705±0.158 µm, n = 79; starved: 1.73±0.115 µm, n = 83, p = 0.9062, two-tailed t-test compared to untreated dataset; DA with food: 1.76±0.145 µm, n = 173, p = 0.7836, two-tailed t-test compared to untreated dataset). However, GLR-1::GFP cluster size was reduced upon starvation conditioning with DA (1.22±0.0688 µm, n = 181, p = 0.000562, two-tailed t-test compared to untreated dataset) and stayed small during the 30 minute recovery phase (Fig. 5c) (1.24±0.0875 µm, n = 118, p = 0.000889, two-tailed t-test compared to untreated dataset). Thus, persistent synaptic remodeling in the VNC is induced in wild-type animals by olfactory associative conditioning but not by food starvation or olfactory adaptation alone.

We next tested if MAGI-1 regulates GLR-1 distribution in the VNC of wild-type and *magi-1(lf)* mutants. In *magi-1(lf)* mutants the size of the GLR-1::GFP clusters was already enlarged in naïve animals and did not decrease after starvation conditioning (Fig. 5d). We then analyzed the size of GLR-1::GFP clusters in conditioned *magi-1(lf)* worms carrying the *Pglr-1::magi-1*, the *Pglr-6::magi-1* or both transgenes together. *Pglr-1::magi-1* alone reduced the size of GLR-1::GFP clusters to the size observed in naïve wild-type animals, but cluster size did not decrease further upon starvation conditioning (Fig. 5d). Expression of MAGI-1 in RIA using the *glr-6* promoter not only restored associative learning (Fig. 3a, d) but also the reduction of GLR-1::GFP cluster size upon starvation conditioning (untreated: 2.143±0.1 µm n = 202; conditioned: 1.473±0.0596 µm, n = 202, p = 1.54*10^−8^, two-tailed t-test compared to untreated dataset). However, GLR-1::GFP cluster size in naïve *Pglr-6::magi-1* animals was still increased similar to non-transgenic *magi-1(lf)* mutants (Fig. 5d) (*magi-1(lf)*: 2.328±0.19 µm n = 85; *magi-1(lf), Ex[Pglr-1::magi-1::gfp]*: 2.143±0.1 µm, n = 202, p = 0.349, two-tailed t-test) Furthermore, GLR-1::GFP clusters in *Pglr-6::magi-1* animals reverted to nearly the size observed in unconditioned animals after 30 minutes of recovery, suggesting that expression of MAGI-1 in RIA alone was not sufficient to mediate a persisting size reduction of GLR-1 clusters at AVA and AVD synapses in the VNC (1.94±0.084 µm, n = 147, p = 5.18*10^−6^, two-tailed t-test compared to conditioned dataset). Finally, all aspects of the conditioned changes in GLR-1 clustering were rescued when *magi-1* was simultaneously expressed in RIA, AVA and AVD using the *glr-1* and *glr-6* promoters (Fig. 5d) (1.3±0.0682 µm, n = 170, p = 5.83*10^−5^, two-tailed t-test compared to untreated dataset). Thus, MAGI-1 plays distinct functions in RIA, AVA and AVD neurons during learning and memory. Expression of MAGI-1 in RIA induces during associative learning the remodeling of GLR-1 containing synapses in AVA and AVD in a cell non-autonomous fashion. At the same time, MAGI-1 acts cell-autonomously in AVA and AVD to consolidate the conditioned changes in GLR-1 clustering during memory formation.

### Efficient memory consolidation requires the HMP-2 β-catenin interaction domain of MAGI-1

**Figure 6:**
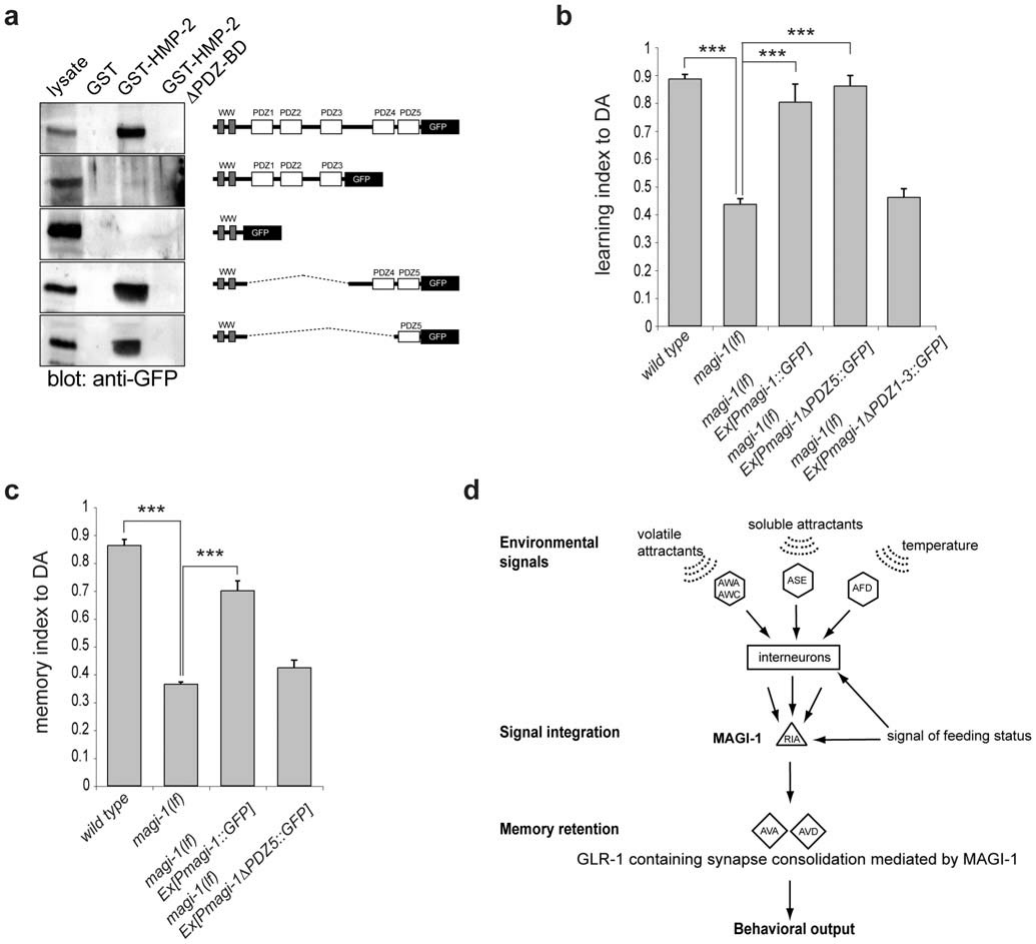
Memory formation requires the MAGI-1-HMP-2 interaction domain. a, Full-length and truncated MAGI-1::GFP proteins (right panels) were incubated with recombinant GST, GST-HMP-2 or GST-HMP-2 lacking the C-terminal PDZ-binding motif (GST-HMP-2 ΔPDZ-BD). Binding of MAGI-1 was detected with an anti-GFP antibody (left panels). *magi-1(lf)* mutant worms carrying a full-length, or truncated (Δ*PDZ5* or Δ*PDZ1-3) magi-1::gfp* minigene were conditioned with DA and tested b, immediately (Learning index = (CI_naïve worms_- CI_DA conditioned worms_)/CI_naïve worms_), or c, following 30 minutes of recovery in the absence of DA (Memory index = (CI_naïve worms_- CI_DA conditioned and recovered worms_)/CI_naïve worms_). All experiments were done in triplicate and repeated at least in two independent experiments. At least two independent transgenic lines were tested for each construct. Error bars indicate average±S.E.M. Datasets were compared to *magi-1(lf)* mutants using two-tailed student's t-test (*** p<0.001). d, Schematic model of the neural circuit controlling associative learning and memory formation in *C. elegans*.

Even though MAGI-1 regulates the dynamic changes in GLR-1 clustering at synapses, we did not detect a direct interaction between MAGI-1 and GLR-1, neither *in vitro* nor *in vivo* (data not shown). Since the vertebrate MAGI protein interacts with β-catenin [3], we tested if *C. elegans* MAGI-1 might bind to the β-catenin homolog HMP-2, the only one of the three *C. elegans* β-catenins that is predicted to contain a C-terminal PDZ binding motif (A.H. and A.S. personal observation). GST pull-down experiments revealed binding of HMP-2 to MAGI-1 (Fig. 6a). This interaction required the PDZ binding motif at the extreme C-terminus of HMP-2 as well as the fifth PDZ domain of MAGI-1 (Fig. 6a). To investigate the physiological significance of the HMP-2/MAGI-1 interaction, we generated a truncated MAGI-1 rescue construct lacking PDZ domain 5 (a deletion of amino acids 954 to 1092 in MAGI-1). The MAGI-1ΔPDZ5::GFP protein localized similar to full-length MAGI-1::GFP (data not shown) and was able to rescue the associative learning defect (Fig. 6b), but neither the memory consolidation nor the nose touch-response defects of *magi-1(lf)* mutants (Fig. 6c and data not shown). In contrast, a deletion mutant of MAGI-1 lacking PDZ1-3 was able to interact with HMP-2 β-catenin (Fig. 6a), but was not sufficient to rescue the associative learning defect of *magi-1(lf)* mutants (Fig. 6b). We conclude that MAGI-1 plays distinct molecular functions in RIA, AVA and AVD neurons, as memory consolidation and nose touch-response but not associative learning require the HMP-2 β-catenin-binding domain in MAGI-1.

## Discussion

In summary, we demonstrate that RIA, AVA, AVD and likely AVE interneurons are responsible for different aspects of associative learning in *C. elegans* (Fig. 6d). While previous data suggested that RIA mediates the association of temperature with feeding status [15], our results show that RIA plays a central role also in olfactory and gustatory associative learning. Thus, RIA may serve to integrate and associate multiple environmental inputs with food starvation. Upon associative conditioning, RIA probably relays a signal to other neurons including the command interneurons AVA, AVD, and likely AVE. Since, there is no direct synaptic connection between RIA and AVA, AVD or AVE interneurons, likely other neurons are also required to mediate signals from RIA to AVA, AVD and AVE. Our results suggest that the multi PDZ-domain protein MAGI-1 is required in RIA for the integration of those environmental inputs during associative learning. On the other hand, memory consolidation, the retention of the conditioned behavior over time, requires the GLR-1 expressing interneurons AVA, AVD and likely AVE, where MAGI-1 is necessary to induce persisting changes in the synaptic GLR-1 cluster size. During associative conditioning, the size but not the number of GLR-1 clusters decrease, which might reflect an increased density of glutamate receptors at post-synaptic membranes. Recently, the long isoform of the *C.elegans* MAGI-1 was demonstrated to influence mechanosensory habituation and GLR-1 receptor degradation through ubiquitination [21]. During habituation the number of GLR-1 positive synapses decrease in the MAGI-1 mutant worms carrying a deletion in the long isoform, suggesting a decrease in glutamate signaling. On the other hand overall number of synapses remains unaffected. In contrast to habituation, associative learning increase synaptic sensitivity and cause a dynamic remodeling of synapses. In our work we demonstrate that complete loss of both isoforms of MAGI-1 impairs mechanosensation through the command interneurons, demonstrated by the lack of response to nose touch in *magi-1(lf)* mutant worms. Furthermore, loss of MAGI-1 has additional effect on neuronal plasticity besides the demonstrated role in non-associative learning [21]. In contrast to the changes observed in the number of GLR-1 punctae during repeated long-term mechano-stimulation, we found that associative learning regulates the average size but not the number of GLR-1 positive synapses. Computational modeling suggested that the glutamate concentration decreases rapidly with increasing distance from the site of release [22], [23]. Hence, slight local shifts in receptor density can lead to large changes in the postsynaptic response. The so-called flexible matrix model describes rapid and continuous changes of the synaptic architecture driven by the actin cytoskeleton [24]. Upon associative learning, a signal from the RIA interneuron induces a transient GLR-1 cluster remodeling in AVA, AVD interneurons that could sensitize the postsynaptic densities. This process is, however, independent of MAGI-1 function in AVA and AVD. On the other hand, the persisting changes in GLR-1 cluster size that correlate with memory consolidation require the presence of MAGI-1 in AVA and AVD. Interestingly, AVA and AVD are backward command interneurons, therefore an increase in synaptic sensitivity in these neurons could be the direct cause of the avoidance behavior upon associative learning. We also show that the same domain in MAGI-1 that is necessary for the interaction with the β-catenin HMP-2 is also required to retain the conditioned behavior over time, but dispensable for associative learning per se. Hence, MAGI-1 could serve as a scaffold and indirectly control glutamate receptor signaling in AVA, AVD and AVE neurons through interaction with the cadherin/catenin complex, for example by the consolidating rearrangement of the actin cytoskeleton and thereby the changes in synapse structure and composition. Further analysis of the role of a MAGI-1/β-catenin complex might give insight into a mechanism of memory formation conserved between *C. elegans* and humans.

## Materials and Methods

### General methods and strains used

Standard methods were used for maintaining and manipulating *C. elegans* [25]. Experiments were conducted at 20°C otherwise indicated. The *C. elegans* Bristol strain, variety N2, was used as the wild-type reference strain in all experiments. Alleles and transgenes used were *magi-1(tm446)*, *nuIs25* [20], *akEx211[glr-3::gfp; glr-3::ICE; lin-15+]; lin-15(n765ts)*, *magi-1(zh66)*, *magi-1(zh66); nuIs25*, *magi-1(zh66); utrEx9[magi-1::gfp, sur-5::mDsRed]*, *magi-1(zh66); utrEx2[pglr-1::magi-1::gfp, sur-5::mDsRed]*, *magi-1(zh66); utrEx3[pglr-6::magi-1::gfp, sur-5::mDsRed]*, *magi-1(zh66); utrEx12[pglr-3::magi-1::gfp, sur-5::mDsRed], magi-1(zh66); utrEx10[pglr-6::magi-1::gfp, pglr-1::magi-1::flag, sur-5::mDsRed]*, *magi-1(zh66); utrEx13[pglr-6::magi-1::gfp, punc-42::magi-1::gfp, sur-5::mDsRed], magi-1(zh66); utrEx14[pglr-6::magi-1::gfp, pnmr-1::magi-1::gfp, sur-5::mDsRed], magi-1(zh66); nuIs25; utrEx4[pglr-1::magi-1::flag, sur-5::mDsRed]*, *magi-1(zh66); nuIs25; utrEx5[pglr-6::magi-1::gfp, sur-5::mDsRed]*, *magi-1(zh66); nuIs25; utrEx6[pglr-6::magi-1::gfp, pglr-1::magi-1::flag, sur-5::mDsRed], magi-1(zh66); utrEx8[magi-1::gfp, snb-1::mDsRed]*, *magi-1(zh66); utrEx17[magi-1*Δ*PDZ5::gfp, sur-5::mDsRed], magi-1(zh66); utrEx19[magi-1*Δ*PDZ1-3::gfp, sur-5::mDsRed]* (all in this work). Transgenic lines were generated by injecting the indicated DNA fragments at a concentration of 50-100 ng/µl into both arms of the syncytial gonad as described [26]. *psur-5::mDsRed* was used as a transformation marker at 10 ng/µl concentration.

### Isolation of magi-1(zh66)

To isolate a deletion mutation in *magi-1*, a library consisting of ∼5*10^5^ mutagenized F1 animals was screened as described [27] with some modifications. Wild-type worms were mutagenized with a combination of 30 µg/ml psoralen (TMP) and 2000 µJ/cm^2^ UV, allowed to recover for 16 hours and five P_0_ animals were plated on each of 960 NGM plates. Cultures were grown and one third of the animals were harvested for DNA isolation. To identify the plate containing the *magi-1(zh66)* deletion, DNA extracts from 12 cultures was pooled and each pool was tested by nested PCR with outer primers OAS-364 [ ^5′^TTCCCGGGTGAAATTGCGACCCAACG TTG^3′^ ], OAS-422 [ ^5′^CAGGATGATGTCCTCATCCTATC^3′^ ], and inner primers OAS-423 [ ^5′^GCTCCGTCGACTAGTTCGAGTAC^3′^ ], OAS-424 [ ^5′^CCATCGGAAGAACTGGTCCAG CTG^3′^ ]. The remaining animals from the culture positive for the *zh66* deletion were subjected to three rounds of sib-selection until a single homozygous *magi-1(zh66)* animal had been identified. Before further analysis, the *magi-1(zh66)* mutation was backcrossed five times against N2 animals.

Olfactory conditioning was assessed as described [17] with some modifications. Starvation conditioning was performed without food in the presence of 2 µl undiluted chemo-attractant spotted on a piece of filter paper and attached to the lid of the plate for 1 hour on 10 cm CTX plates (5 mM KH_2_PO_4_/K_2_HPO_4_ pH = 6.0, 1 mM CaCl_2_, 1 mM MgSO_4_, 2% agar). Naïve and conditioned worms were given a choice between a spot of 0.1% (vol/vol) DA or IA in ethanol with 20 mM sodium-azide and a counter spot with ethanol and sodium-azide. After one hour the animals were counted and a chemotaxis index was calculated as described [28].

Chemotaxis to water-soluble compounds was assessed as described with some modifications [29]. Pairs of opposite quadrants of four-quadrant Petri plates (Falcon X plate, Becton Dickinson Labware) were filled with buffered agar (2% agar, 5 mM KH_2_PO_4_/K_2_HPO_4_ pH 6.0, 1 mM CaCl_2_ and 1 mM MgSO_4_), either containing 25 mM NaCl or no salt. Adjacent quadrants were connected with a thin layer of molten agar. A population of well-fed, young adults was washed three times with CTX buffer (5 mM KH_2_PO_4_/K_2_HPO_4_ pH 6.0, 1 mM CaCl_2_ and 1 mM MgSO_4_) and 100−200 worms were placed at the intersection of the four quadrants. The distribution of the worms over the four quadrants was determined after 10 minutes. For NaCl conditioning, animals were exposed to 25 mM NaCl in CTX buffer for 4 hours.

### Thermotaxis assay

A steep thermal gradient on a thin agar plate was established as described previously [13]. After build up of the gradient, plates were separated into “20°C”, “25°C” and “15°C” regions where “20°C” corresponded to the 19°C to 21°C zone, “25°C” to a warmer and “15°C” to a cooler region. For conditioning, animals were grown at the indicated temperature for 2 days and starved for 4 hrs before testing. The numbers of worms in each of the three temperature zones were determined after 10 minutes, and the thermotaxis was calculated as described [15].

Locomotory rate assays were performed on a bacterial lawn as described [14], [30]. Briefly, worms were grown under uncrowded conditions with or without food on conditioning plates for 1 hour. Two minutes after transfer on 6 cm assay plates seeded with OP50, the number of body bends was counted for 1 minute for 8 animals from each strain.

### Microscopy

GFP (or GFP variants)-tagged proteins were detected with a Zeiss Axiovert 200M LSM 5 Pascal confocal microscope. Animals were immobilized with sodium-azide and GLR-1::GFP was recorded posterior to the vulva, along the z-axis. Quantification of GLR-1::GFP cluster size was performed on the projected z-sections using Openlab 5.0 software package (Improvision).

### Molecular biology

Promoter-gene fusions were generated by PCR fusion as described [31]. All fragments were amplified using proof reading polymerase (LA Taq, Takara) from *C. elegans* genomic DNA. Primer sequences are available on request. GST-fusion proteins were expressed in *E. coli* and purified on glutathione sepharose using standard protocols. After incubation with total worm protein extracts prepared by lysing worms in lysis buffer (50 mM Tris-HCl pH 7.4, 1% Triton-X 100, 10% glycerol, 150 mM NaCl, 1 mM NaF, 1 mM Na-orthovanadate, and protease inhibitor coctail), bound MAGI-1::GFP proteins were detected on Western blots using anti-GFP antibodies (Roche).

## Supporting Information

Figure S1 (0.30 MB DOC)

Figure S2 (0.13 MB DOC)

Figure S3 (0.20 MB DOC)
