# Supplementary material for: Neuron-Specific Regulation of Associative Learning and Memory by MAGI-1 in C. elegans
Source: PLoS One. 2009 Jun 24;4(6):e6019. doi: 10.1371/journal.pone.0006019 (PMC2696103; doi:10.1371/journal.pone.0006019)
Supplement: Figure S1 — (0.30 MB DOC) [file pone.0006019.s001.doc]

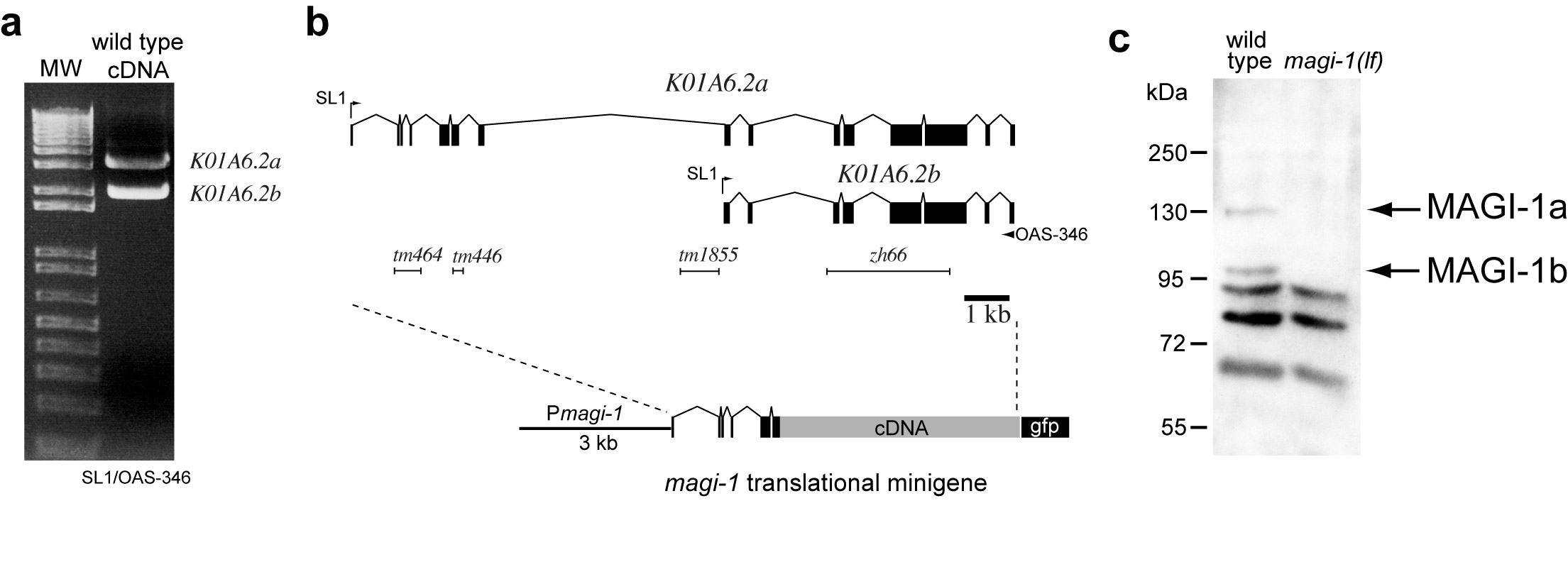


Figure S1. The *magi-1* locus encodes two alternative transcripts. a, RT-PCR performed from wild-type cDNA with SL1 and a *magi-1* gene-specific primer shown in panel b. b, illustration of the genomic structure of the transcripts, position of deletions and the structure of the rescuing *magi-1::gfp* minigene. c, Western-blot analysis of total protein extract from wild-type and *magi-1(lf)* worms using MAGI-1 specific antibody. Arrows indicate the two MAGI-1 isoforms at 130 kDa and 97 kDa, respectively.
