# Supplementary material for: Neuron-Specific Regulation of Associative Learning and Memory by MAGI-1 in C. elegans
Source: PLoS One. 2009 Jun 24;4(6):e6019. doi: 10.1371/journal.pone.0006019 (PMC2696103; doi:10.1371/journal.pone.0006019)
Supplement: Figure S2 — (0.13 MB DOC) [file pone.0006019.s002.doc]

Figure S2. Naïve chemotaxis and sensory modulation of the locomotory rate in wild-type and *magi-1(lf)* mutants. a, chemotaxis towards the indicated chemicals was tested in wild-type and *magi-1*
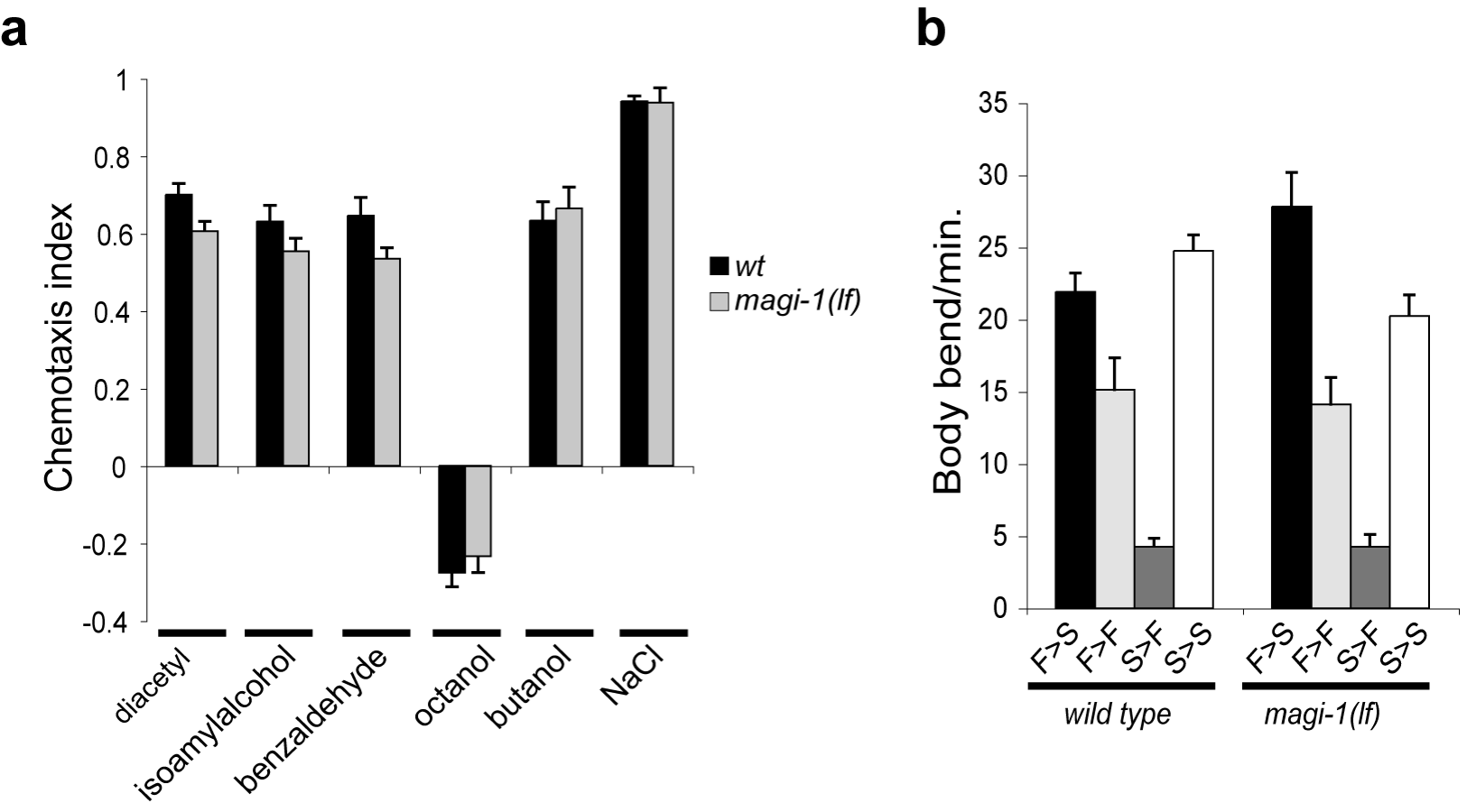
*(lf)* worms as described [28]. b, Locomotory rate of fed (F) or starved worms (S) was tested on NGM plates in presence (>F) or absence of food (>S) (n=8).
