# Supplementary material for: Neuron-Specific Regulation of Associative Learning and Memory by MAGI-1 in C. elegans
Source: PLoS One. 2009 Jun 24;4(6):e6019. doi: 10.1371/journal.pone.0006019 (PMC2696103; doi:10.1371/journal.pone.0006019)
Supplement: Figure S3 — (0.20 MB DOC) [file pone.0006019.s003.doc]

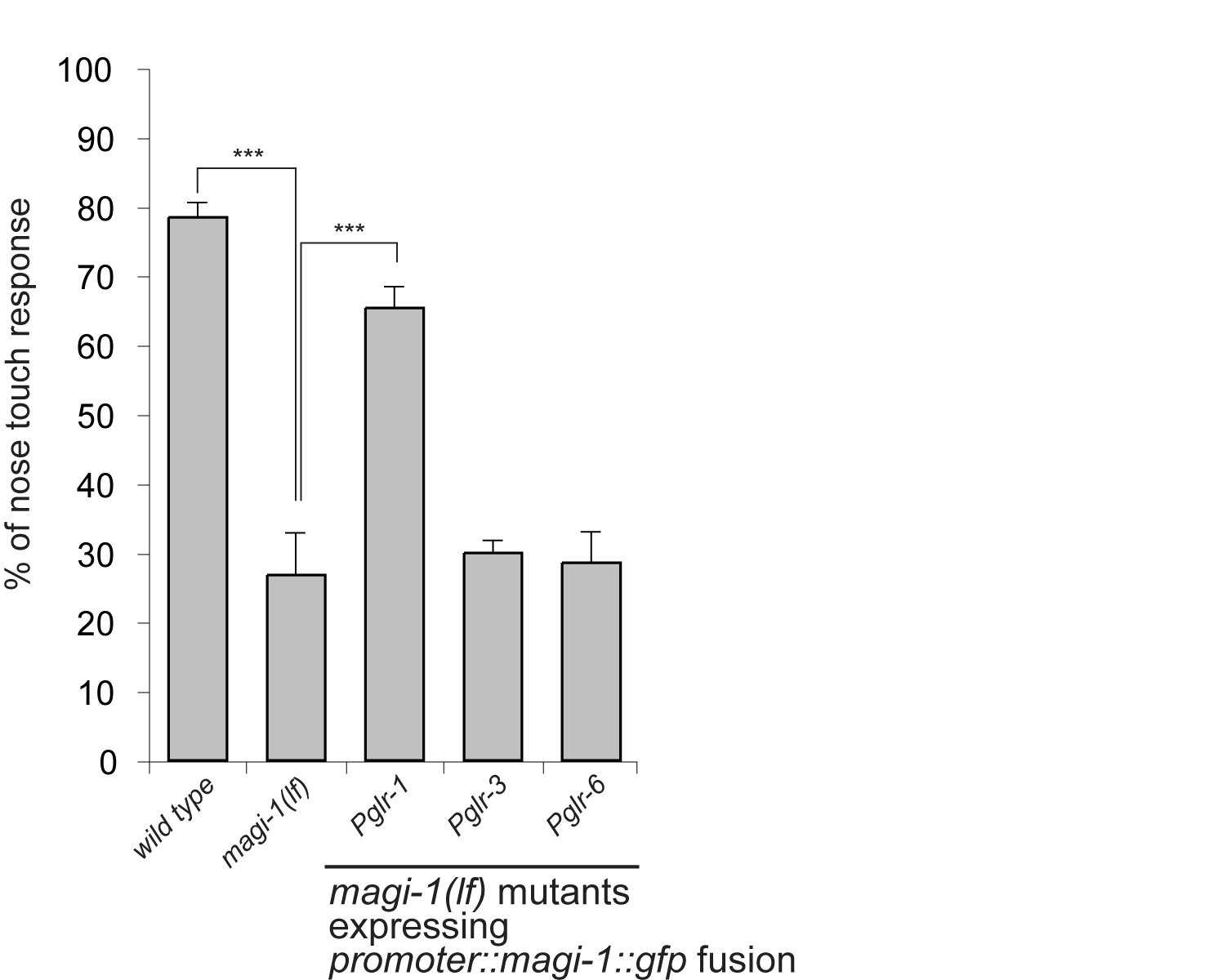


Figure S3. MAGI-1 is required for proper nose-touch response. Avoidance of light touch to the nose was tested as described [32]. The number of trials were: wild type= 199, *magi-1(lf)*= 202, *magi-1(lf); Ex[glr-1::magi-1]*= 244, *magi-1(lf); Ex[glr-3::magi-1]*= 63, *magi-1(lf); Ex[glr-6::magi-1]*= 105. Three independent tests were quantified, and three independent transgenic lines were tested for each construct. Error bars indicate average ± S.E.M. Significance between datasets as indicated was tested with two-tailed t-test (*** p< 0.001).
